# Impact of air temperature variation on the ixodid ticks habitat and tick-borne encephalitis incidence in the Russian Arctic: the case of the Komi Republic

**DOI:** 10.1080/22423982.2017.1298882

**Published:** 2017-03-31

**Authors:** N. Tokarevich, A. Tronin, B. Gnativ, B. Revich, O. Blinova, B. Evengard

**Affiliations:** ^a^ Laboratory of Zoonoses, St Petersburg Pasteur Institute, St Petersburg, Russia; ^b^ Scientific Research Center for Ecological Safety, Russian Academy of Sciences, St Petersburg, Russia; ^c^ Center for Hygiene and Epidemiology of Komi Republic, Syktyvkar, Russia; ^d^ Institute of Forecasting, Russian Academy of Sciences, Moscow, Russia; ^e^ University Hospital, Umea University, Umea, Sweden

**Keywords:** Tick-borne encephalitis, climate change, *I. persulcatus*, NDVI, Arctic, Komi Republic

## Abstract

**Background**: The causes of the recent rise of tick-borne encephalitis (TBE) incidence in Europe are discussed. Our objective was to estimate the impact of air temperature change on TBE incidence in the European part of the Russian Arctic.

**Methods**: We analysed the TBE incidence in the Komi Republic (RK) over a 42-year period in relation to changes in local annual average air temperature, air temperature during the season of tick activity, tick abundance, TBE-prevalence in ticks, tick-bite incidence rate, and normalised difference vegetation index within the area under study.

**Results**: In 1998–2011 in RK a substantial growth of TBE virus (TBEV) prevalence both in questing and feeding ticks was observed. In 1992–2011 there was 23-fold growth of the tick-bite incidence rate in humans, a northward shift of the reported tick bites, and the season of tick bites increased from 4 to 6 months. In 1998–2011 there was more than 6-fold growth of average annual TBE incidence compared with 1970–1983 and 1984–1997 periods. This resulted both from the northward shift of TBE, and its growth in the south. In our view it was related to local climate change as both the average annual air temperature, and the air temperature during the tick activity season grew substantially. We revealed in RK a strong correlation between the change in the air temperature and that in TBE incidence. The satellite data showed NDVI growth within RK, i.e. alteration of the local ecosystem under the influence of climate change.

**Conclusions**: The rise in TBE incidence in RK is related considerably to the expansion of the range of *Ixodes persulcatus*. The territory with reported TBE cases also expanded northward. Climate change is an important driver of TBE incidence rate growth.

## Introduction

Tick-borne infections are an increasing burden on healthcare in many countries. In the EU in 2012 tick-borne encephalitis (TBE) was included in the list of notifiable diseases,[1] and since then it is notifiable in 17 European countries.[2] In some regions of northern Europe there has been an uptrend in TBE cases.[3–6]

Immediate causes of TBE incidence growth and the distribution of Ixodidae ticks, the main vector of TBE virus (TBEV), are still not understood and depend on a number of factors,[7–12] the impact of climate change being one of them.[13–15]

TBE incidence depends on a number of factors, air temperature being one of them. Therefore it is necessary to study the impact of air temperature at the boundary of the region inhabited by TBE vectors (ixodid ticks), as both air temperature and TBE incidence have increased in recent years in the north of European Russia.

**Figure 1.**
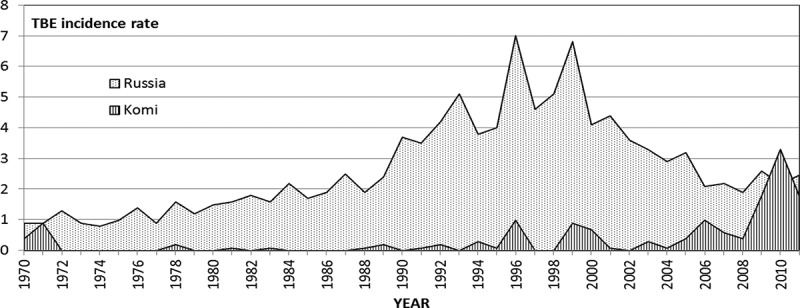
TBE incidence rate in Russia and in RK.

Earlier we have shown that the increase in average annual air temperature and “effective” temperature (in the period of tick activity) has a significant impact on the northward expansion of ixodid ticks and the rise in TBE incidence in Arkhangelsk Oblast of Russia.[16] Our studies revealed also that within the same period local precipitations varied very slightly and provided no significant impact on the ecology of ticks. In this regard, one may assume that events in Komi Republic (RK), neighbouring Arkhangelsk Oblast, may be influenced by the same climatic factors. Indeed, there has been a rise in TBE incidence and tick-bite incidence rates in RK (Figure 1). However, “the association between those ups and changing climatic conditions require additional research and observation” (p. 50).[17] Therefore, as part of an international collaboration on effects of climate change on the spread of infectious diseases in the north (Clinf) we designed a study where we made use of RK official statistics, geographic and demographic data published by Russian Federation Federal State Statistic Service [see Rosstat http://www.gks.ru/], climatic, satellite-provided data for the analysis of vegetation change over time, and ecological and epidemiological data.

**Figure 2.**
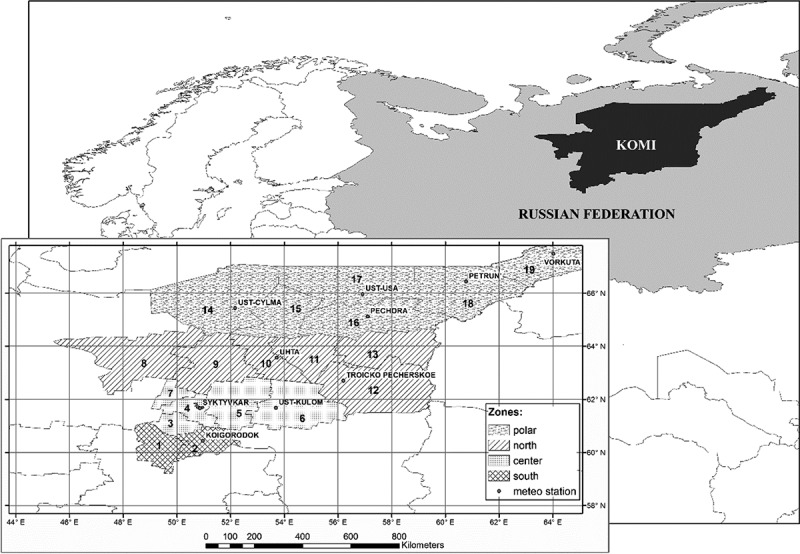
Komi Republic in the Russian Federation. Cutaway: administrative districts by zones. Southern zone: 1, Priluzskiy (S1); 2, Koigorodskiy (S2). Central zone: 3, Sysolskiy (C3); 4, Syktyvdinskiy + Syktyvkar city (C4); 5, Korterosskiy (C5); 6, Ust-Kulomskiy (C6); 7, Ust-Vymskiy (C7). Northern zone: 8, Udorskiy (N8); 9, Knyajpogostskiy (N9); 10, Ukhtinskiy (N10); 11, Sosnogorskiy (N11); 12, Troitsko-Pechorskiy (N12); 13, Vuktylskiy (N13). Polar zone: 14, Ust-Tsilemskiy (P14); 15, Ijemskiy (P15); 16, Pechorskiy (P16); 17, Usinskiy (P17); 18, Intinskiy (P18); 19, Vorkutinskiy (P19).

RK consists of 20 administrative units: 19 rural districts and one city district (Syktyvkar); however, Syktyvkar inhabitants mostly acquired TBEV in the adjacent Syktyvdinsk district, and for the ease of data processing we considered those two units together. About 95% of RK territory is taiga, and 5% tundra. For the purpose of this study 19 districts were divided into four geographic zones: Polar zone (polar tundra and tundra forest belt); Northern zone (boreal taiga), Central zone (middle taiga), Southern zone (southern taiga) (Figure 2).

The RK population was 926,800 (1 January 2011), including the native Komi population, and minor indigenous ethnic groups: Khanty, Mansi and Nenets. Between 1970 and 2011 the RK population decreased by 4%.

Most of the Komi territory is covered by taiga (about 89%), with the far north-east occupied by tundra (2%). Other vegetation is represented by intermediate forms, swamps and meadows. Around 32,800 km^2^ of mostly boreal forest (as well as some alpine tundra and meadows) in the Republic’s Northern Ural Mountains have been recognised in 1995 as a UNESCO World Heritage site, Virgin Komi Forests. It is the first natural UNESCO World Heritage site in Russia and the largest expanse of virgin forests in Europe. Siberian spruce, Scots pine and birch are widespread in this area.

The climate of RK is cold continental, subarctic, class “Dfc” by the Köppen climate classification.[18] Annual air temperature from 1970 to 2013 is about −1.3°С. Total precipitation is in the range of 600–700 mm per year, evaporation is about 200–300 mm per year, so the climate is humid with more precipitation than evaporation.

## Materials and methods

### Environmental and epidemiological data

The information used in our assessment of TBE situation in RK was as follows.

The monthly data on tick abundance (ticks per flag per hour) were calculated starting from the collection of ticks flagged (flannel flag sized 1.0 × 0.6 m) from vegetation in S1, S2, C3, C4, C6 administrative districts (see Figure 2) from May to July (*I. persulcatus* activity season) in 1970–1971, 1974–1980, 1982–1984, 1986–1992, 2000–2003, 2005–2011). Ticks were gathered in dull calm days when the air temperature was 12–18°С, and humidity 70–90%. Every 5 min ticks were removed from the flag with a surgical forceps, placed individually into a glass vial, and delivered to the laboratory the same day.

The standard method [19] was applied for tick species identification.

The collected ticks were tested for TBEV using enzymoimmunoassay (Vecto VKE-IgG manufactured by Vector-best, Novosibirsk, Russia).

The number of RK inhabitants seeking medical care after tick bites in 1992–2011 was considered. The background information including date and place of the event was provided to Russian Federal Service for Surveillance on Consumer Rights Protection and Human Wellbeing (Rospotrebnadzor) by medical institutions of each RK district, and the tick-bite incidence rate (TBIR), i.e. number of tick victims per 100,000 of population, was calculated as follows:

(1) 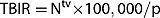

where N^tv^ is number of tick victims within the district under study during the year, and р is the number of the district inhabitants.

TBE incidence rate in 1970–2011 was calculated as follows:

(2) 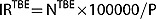

where IR^TBE^ is TBE incidence rate, N^TBE^ is the number of TBE cases within the district under study during the year, and р is the number of the district inhabitants.

TBE was diagnosed on the basis of clinical and epimediological data, and usually (97.3%) confirmed by seroprevalence study of paired sera in the dynamics of infectious process with certified diagnostic preparations.

Our study covered only cases of tick bites and/or TBEV infection occurring within RK territory, the criterion being the production of evidence that the patient had not left the district of residence at least 1 month prior to the event. In Russia, as in the former Soviet Union, health education firmly stressed that a person should rapidly seek healthcare after tick bite. The tick was then analysed for TBE and *Borrelia burgdorferi*. Specific immunoglobulin was offered if the tick contained TBEV. This explains the high number of persons with tick bites seeking healthcare.

### Meteorological data

Meteorological data (air temperature in 1960–2013) were obtained from two main sources: National Climatic Data Center, USA (http://www.ncdc.noaa.gov/) and the Russian World Data Center for Meteorology (http://www.meteo.ru/), the data being collected at 10 meteorological stations in RK (Table 1). Two types of database products were used: monthly-averaged and daily-averaged air temperatures. To calculate the effective temperatures we used data on mean daily temperatures reported by three stations: Koigorodok (Southern zone), Troicko-Pecherskoe (Northern zone), and Syktyvkar (Central zone).

**Table 1.** RK meteorological stations.

Name	WMO number	Latitude	Longitude	RK zone	Date range, years
Vorkuta	23226	67°29′N	64°01′E	Polar	1973–2013
Petrun’	23324	66°26′N	60°46′E	Polar	1960–2013
Ust’-Usa	23412	65°58′N	56°55′E	Polar	1960–2013
Ust’-Cil’Ma	23405	65°26′N	52°10′E	Polar	1900–2013
Pechora	23418	65°07′N	57°06′E	Polar	1960–2013
Troicko-Pecherskoe	23711	62°42′N	56°12′E	Northern	1900–2013
Uhta	23606	63°34′N	53°43′E	Northern	1973–2013
Ust’-Kulom	23803	61°41′N	53°41′E	Central	1973–2013
Syktyvkar	23804	61°40′N	50°51′E	Central	1900–2013
Koigorodok	23904	60°26′N	50°58′E	Southern	1960–2013

The average annual air temperature (AAAT) was calculated from the average monthly values reported by each station.

(3) 
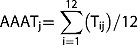

where T_ij_ is monthly average air temperature on station j in month i. This way AAAT_j_ for all station were obtained. The AAAT values for Polar, Northern, Central, Southern zones, and for RK as a whole were defined as the average of AAAT values reported by the stations of the corresponding zone.

### Satellite data

The normalised difference vegetation index (NDVI) derived from satellite-provided data was used to assess the change in vegetation during the period under study.[20]

(4) 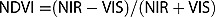

where VIS and NIR are the spectral reflectance measurements acquired by satellite sensors in the visible (red) and near-infrared regions, respectively. Calculations of NDVI for a given point result in a number that ranges from minus one to plus one. However, lack of vegetation gives a value close to zero or below. NDVI values close to +1 (0.8–0.9) indicates the highest possible density of green leaves.

Two satellite-based databases covering the time period from 1982 to 2010 (Table 2) were used: NOAA (AVHRR) data in range 1982–2001 with spatial resolution 8 km,[21] and Terra (MODIS) data in range 2000–2010 with resolution of 0.05° (about 5 km for the Komi area) [https://lpdaac.usgs.gov/products/modis_products_table/mod13c1].

**Table 2.** Databases used for NDVI assessment.

Satellite (instrument)	Data range, years	Spatial resolution	Database
NOAA (AVHRR)	1982–2001	8 km	GIMMS (Global Inventory Modeling and Mapping Studies)
Terra (MODIS)	2000–2010	0.05°	NASA Land Processes Distributed Active Archive Center, product MOD13C1

### Statistical analysis

The primary statistical data processing resulted in values averaged over time intervals, and the standard deviations of the variables under study.

Pearson correlation analysis was performed to assess the correlation between the air temperature and TBE incidence.

(5) 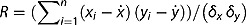

where x_i_ indicates values from dataset {x_1_, …, x_n_} containing n values; y_i_ indicates values from dataset {y_1_, …, y_n_} containing n values; ẋ and ẏ are the sample mean for dataset x and analogously for y; δ_x_ and δ_y_ are the standard deviation of x and y respectively. Microsoft Excel was used for correlation assessment (Microsoft).

Regression analysis was applied to determine the AAAT that triggers TBE incidence growth. It was conducted to evaluate the statistical significance of the results. Microsoft Excel was used for regression analysis.

The sum of effective temperatures reflects the amount of heat necessary for full development of all stages of *Ixodes persulcatus* in its season of activity, and was calculated by the formula [22,23]:

(6) 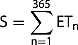

where S is the annual sum of effective temperatures; ET_n_ = (t_n_ – t_min_) indicates effective temperature at day n; t_min_ is temperature limit, °С (+10°С for *Ixodes persulcatus*); and t_n_ is day “n” average temperature, °С (only if t_n__ _> t_min_).

## Results

### Tick species, abundance, and TBE prevalence

The maximal abundance of ixodid ticks was registered in July. To determine the dominant species 241 ticks at random were examined, of which 225 (93.4 ± 1.6%) proved to be *Ixodes persulcatus*. In July according to long-term observations the tick abundance in the Southern zone (S1, S2) was 2.3–3.2 ticks (imago) per flag per hour, and in the Central zone (С3, C4, C6) it was 0.2 (Table 3).

**Table 3.** Geographical coordinates of sites where ticks were flagged.

	Latitude, N	Longitude, E
S1 (Priluzskiy)	60°7′46″–60°41′6″	48°43′51″–49°42′49″
S2 (Koigorodskiy)	60°26′43″–60°40′8″	50°21′20″–50°59′59″
C3 (Sysolskiy)	60°50′49″–61°5′12″	49°38′17″–50°20′21″
C4 (Syktyvdinskiy + Syktyvkar city)	61°9′29″–61°57′7″	50°10′42″–50°50′9″
C6 (Ust-Kulomskiy)	61°15′33″–61°48′48″	53°10′56″–53°51′40″

In 1998–2008 in Southern and Central zones (S1, S2, C3, and C4) 133 questing ticks flagged from plantation were tested, and TBEV was found in none of them.

In 2009–2011 in the same sites 285 ticks were flagged and tested, and 27 (9.5%) proved to be TBEV-positive.

In 1998–2008, 1,968 feeding ticks were collected from humans and domestic animals (dogs and cats). In average 6.3 ± 4.5% of those ticks were TBEV-positive. TBEV-positive ticks belonged to collections from eight districts: S1, S2, C3, C4, C5, C6, C7 and N9.

In 2009–2011, within the same districts, 4,080 feeding ticks were collected from humans and domestic animals; TBEV-prevalence in them was higher: 11.0 ± 1.6% (p-value 0.016) (Table 4).

**Table 4.** TBE virus prevalence in ticks removed from humans and domestic animals in 1998–2008 and in 2009–2011.

RK district	Positive /studied (%)	
	1998–2008	2009–2011
N9	3/53 (5.7%)	13/129 (10.1%)
С7	4/76 (5.2%)	20/193 (10,3%)
С6	19/210 (9.1%)	30/310 (9.7%)
С5	7/137 (5.1%)	21/210 (10.0%)
С4	35/656 (5.3%)	159/1296 (12.3%)
C3	27/433 (6.2%)	94/894 (10.5%)
S2	9/126 (7.1%)	27/290 (9.3%)
S1	20/277(7.2%)	94/758 (12.4%)
**Total**	**124/1968 (6.3%)**	**449/4080 (11.0%)**

### Tick bite incidence rate (TBIR) in RK

In 1992–2011 in RK there was 23-fold increase in number of patients seeking medical care because of tick bites: from 103 in 1992 to 2,369 in 2011.

In 1992–2001 TBIR was 23, while in 2002–2011 it reached 110. The most pronounced increase in TBIR, up to 228, was observed in 2009–2011.

**Figure 3.**
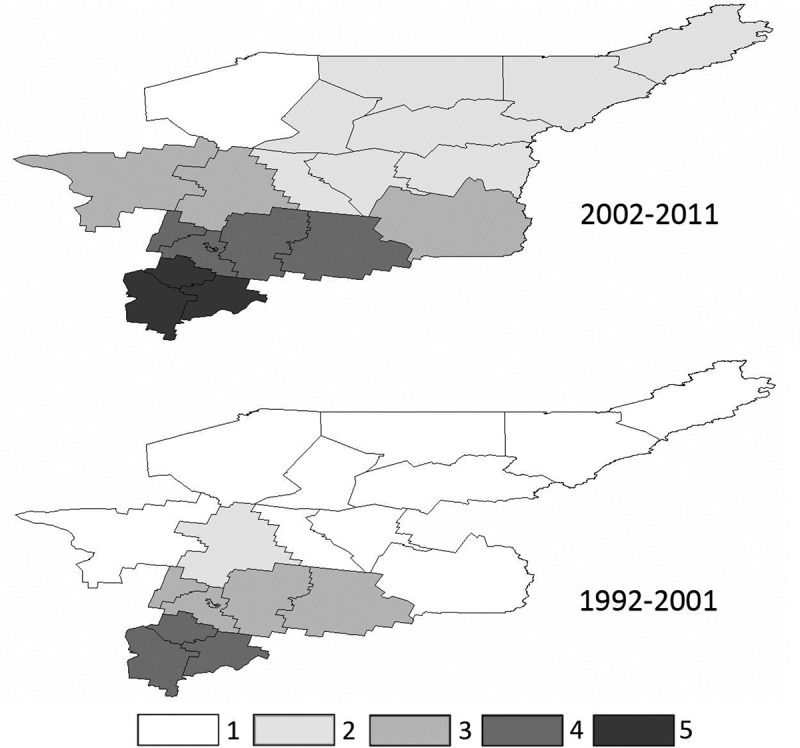
Tick bite incidence rate in RK districts in 1992–2001, and in 2002–2011. 1, < 0.1; 2, 0.1–10; 3, 10–100; 4, 100–1,000; 5, > 1,000.

The boundary of territory with recorded tick bites shifted northward considerably (Figure 3, Table 5). In 1992–1993 tick bites occurred only in Southern (S1, S2), and Central districts (C3, C4), but in 2006–2011 they were recorded in all RK districts, except for P14.

**Table 5.** TBIR (tiсk bite incidence rate) in RK districts in 1992–2001, and in 2002–2011.

RK district	1992–2000	2001–2011
P19	0.00	0.16
P18	0.00	0.45
P17	0.00	1.07
P16	0.00	1.60
P15	0.00	1.84
P14	0.00	0.00
N13	0.00	6.59
N12	0.00	25.42
N11	0.00	4.77
N10	0.00	6.66
N9	5.38	69.01
N8	0.00	11.77
C7	14.04	181.04
C6	32.79	221.29
C5	30.89	154.26
C4	20.75	101.09
C3	188.34	1071.08
S2	272.47	1024.07
S1	326.49	1185.50

The season of tick activity extended from 4 to 6 months: in 1992–2001 tick bites occurred from May to August, while in 2011 they were recorded from April to September.

Most of those tick bites occurred during recreation time in the forest (51.7%), allotment gardens (33.6%), parks or cemeteries (8.9%); only 5.8% of tick bites occurred during working time or at an unknown place (Table 6).

**Table 6.** Places of tick bites in RK.

Place of tick bite	Tick bite incidence	
	Number of tick-bitten humans	%
Recreation areas, forests	3,114	51.7
Allotment gardens	2,023	33.6
Parks or cemeteries	536	8.9
Work areas or unknown areas	349	5.8
Total	6,023	100

### TBE incidence rate

For clarity of presentation the analysed 42-year time range (1970–2011) was divided into three 14-years periods: I: 1970–1983, II: 1984–1997, and III: 1998–2011.

**Figure 4.**
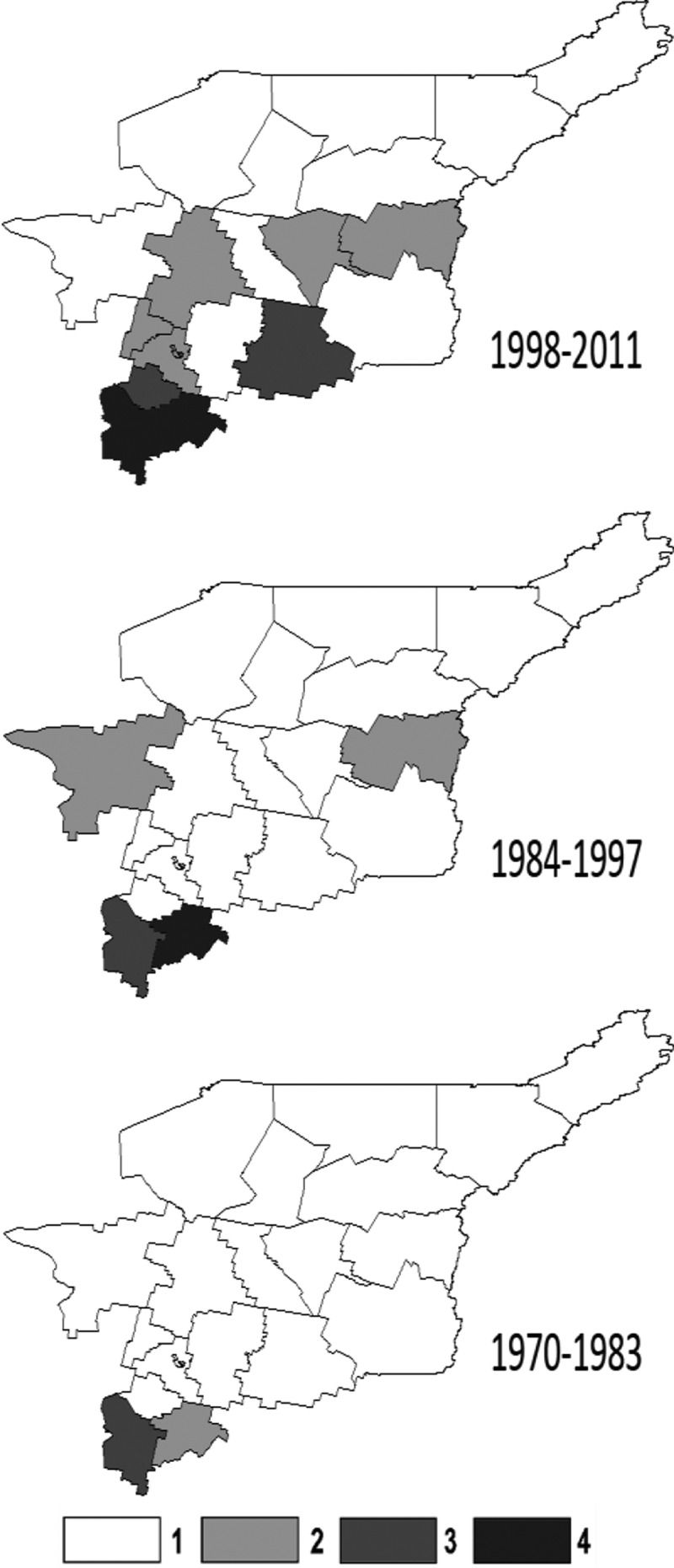
TBE incidence rate in RK districts for 14-year periods: 1, 0.0–0.099; 2, 0.1–0.99; 3, 1.0–4.99; 4, > 5.0.

In 1970–2011 135 TBE cases were reported in RK. There was a clearly increasing trend of TBE incidence (Figures 1 and 4, Table 7). Thus, the average annual TBE incidence rate was virtually the same in periods I (0.12) and II (0.11), but underwent a 6-fold increase in period III (0.74), and grew dramatically in 2009–2011 (2.17). This trend was opposite to the decreasing trend in Russia as a whole within the same time periods. (Table 8)

**Table 7.** TBE incidence rate in RK districts for three 14-year periods

RK district	1970–1983	1984–1997	1998–2011
P19	0.00	0.08	0.00
P18	0.00	0.00	0.00
P17	0.00	0.00	0.00
P16	0.00	0.09	0.09
P15	0.00	0.00	0.00
P14	0.00	0.00	0.00
N13	0.00	0.26	0.27
N12	0.00	0.00	0.00
N11	0.00	0.00	0.14
N10	0.00	0.00	0.06
N9	0.00	0.00	0.31
N8	0.00	0.41	0.00
C7	0.00	0.00	0.36
C6	0.00	0.00	2.84
C5	0.00	0.00	0.00
C4	0.00	0.00	0.59
C3	0.00	0.00	4.32
S2	0.59	6.11	9.84
S1	3.20	1.74	14.66

**Table 8.** TBE incidence rate in RK and in whole Russia

Time period	TBE incidence rate in RK	TBE incidence rate in Russia
1970–1983	0.12	1.24
1984–1997	0.11	3.46
1998–2011	0.74	3.38
2009–2011	2.17	2.43

The boundary of territory with recorded TBE cases moved northward considerably in recent years. During Period I TBE cases occurred only in RK Southern zone (S1, S2), during Period II in Southern (S1, S2) and Northern (N8, N13) zones, and in Period III in all zones (S1, S2, C3, C4, C6, C7, N9, N11, N13). A few TBE cases occurred even in N10, and P16. The sharp TBE incidence rise in RK resulted both from a northward shift of TBE cases and from their more frequent occurrence in Central and, especially, Southern zones (Figure 4).

### Changes in temperature and TBE incidence rate

In 1970–1989 in RK the AAAT ranged from −3.5°C to +0.9°C, and averaged −1.6°C. In 1990–1999 AAAT was −1.35°C on average in the same range from −3.5°C to +0.9°C.

**Figure 5.**
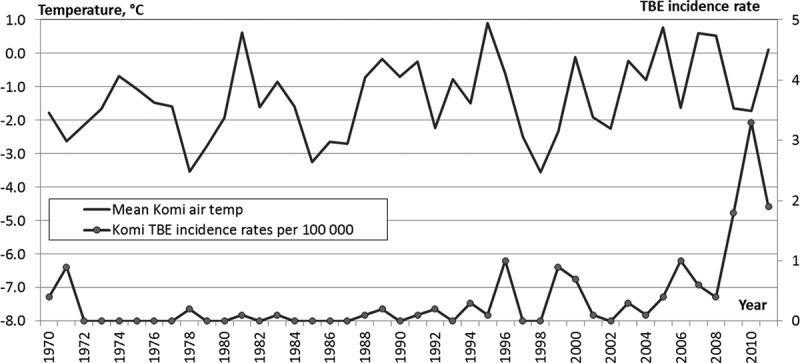
AAAT and TBE incidence rate in RK.

After 2000 AAAT in RK increased gradually and never fell below −2.2°C. In 2000–2011 AAAT was −0.7°C in range of −2.2°C to +0.8°C, i.e. exceeded the level of 1970–1989 by 0.9°C. TBE incidence rate increased with AAAT (Figure 5).

**Figure 6.**
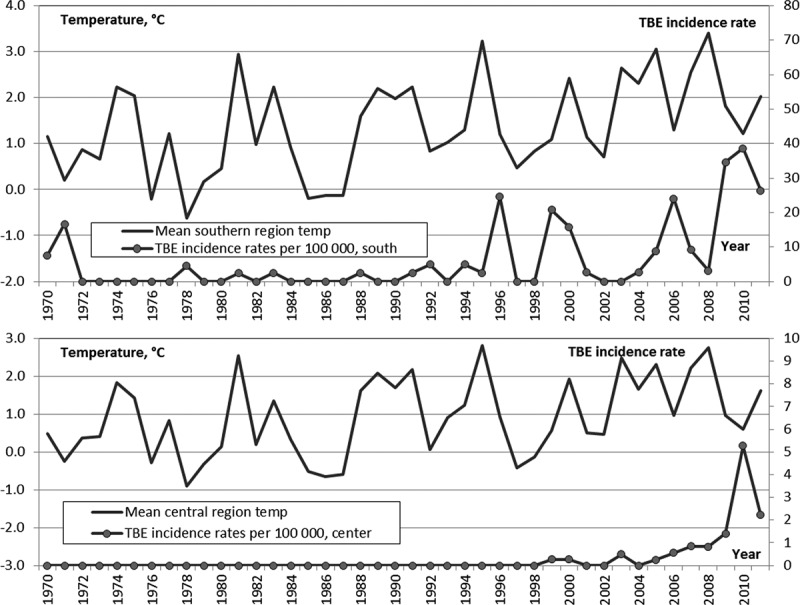
AAAT and TBE incidence rate in Southern (top) and Central (bottom) zones of RK.

In the Southern zone AAAT (Figure 6) increased from +0.9°C (1970–1989) to +2.0°C (2000–2011). The TBE incidence rate remained low in the 1980s, but it started growing after 1990 to reach 38 in 2010).

Within the same period in the Central zone AAAT (Figure 6) increased from +0.5 to +1.5°C. Here the TBE incidence rate steadily rose after the end of the 1990s and exceeded 5 by 2010.

In the Northern zone in 1970–1989 AAAT was −0.7°C, but in 2000–2011 it reached +0.1°C. Only sporadic TBE cases were reported here during the entire analysed period.

To reveal the dependence of the TBE incidence rate on climate change we considered the longest series of meteorological data (Table 1). AAAT was calculated and its averaging was performed by the moving-average method with a 9-year window. So the long series of meteorological observations allowed us to properly compare the averaged air temperatures with TBE incidence rates also averaged with a 9-year window.

**Figure 7.**
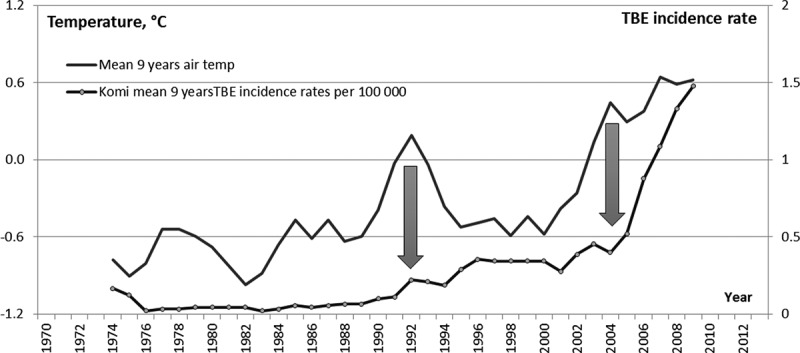
AAAT and TBE incidence in RK averaged with 9-year window. The blue arrows show the periods of sharp rise in the air temperature.

The comparison (Figure 7) demonstrated that the significant increase in AAAT between 1989 and 1995 resulted in a rise of TBE incidence rate to 0.25. The second period of AAAT growth that started in 2000 resulted in a dramatic, up to 1.5, increase in the TBE incidence rate.

**Figure 8.**
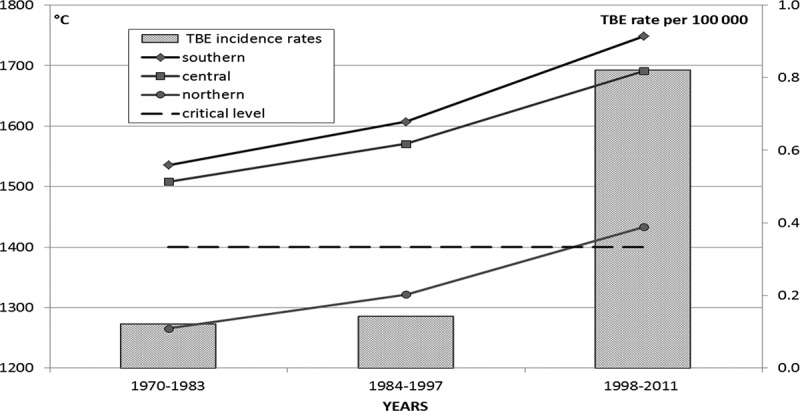
Annual sums of effective temperatures averaged over 14-year periods for Northern, Central, and Southern zones of RK, and annual average TBE incidence rate for RK.

The sum of effective temperature trends (Figure 8) calculated for the 14-year periods revealed significant increment in the sum of effective temperatures between 1970 and 2000: it increased by 213°C in the Southern zone, by 183°C in the Central zone, and by 168°C in the Northern zone.

An analysis of the relationship between the changes in AAAT and TBE incidence rate within each RK zone (Table 9) shows high probability of a strong correlation coefficient between them in the whole RK 0.77 (p<0.0001) and in its Northern, Central and Southern zones separately. The strength of the correlation declines from Southern to Northern zone, and in the Polar zone it is not statistically significant.

**Table 9.** Correlation analysis of AAAT and TBE incidence rate in RK in 1970–2011. The data are smoothed with 9-year window.

Zone	Pearson’s correlation coefficient	Confidence interval	p-value
RK as whole	0.77	0.70–0.84	<0.0001
Polar zone	0.16	−0.02–0.34	0.0855
Northern zone	0.61	0.49–0.72	<0.0001
Central zone	0.66	0.55–0.76	<0.0001
Southern zone	0.86	0.81–0.91	<0.0001

**Figure 9.**
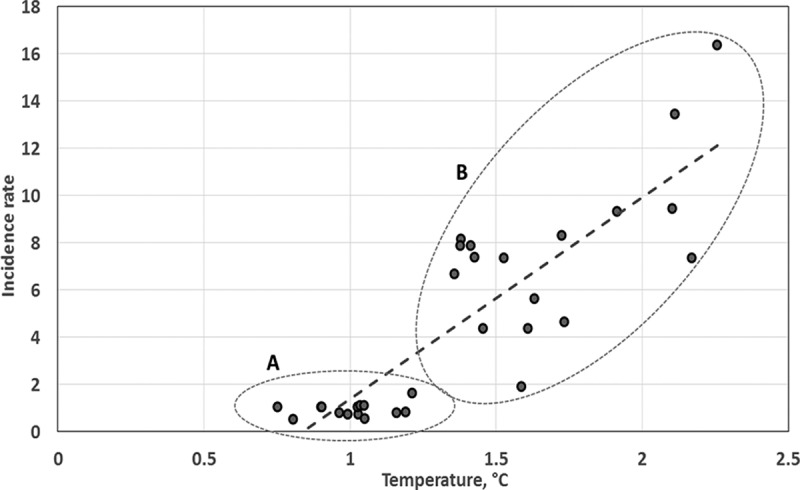
The regressional analysis of TBE incidence rate and AAAT in RK Southern zone in 1970–2011. Both AAATs and TBE incidence rates are averaged with 9-year window. The dash line shows the linear trend. “A”-balloon displays the stagnation period (low AAAT, low TBE incidence rate), “B”-balloon displays the uptrend in AAAT and TBE incidence rate.

To estimate the regression dependence we used AAAT and TBE incidence rates for the Southern zone averaged by 9-year intervals (Figure 9). It was shown that the set of data may be divided as follows: a stagnation region when AAAT rose from 0.75 to 1.2°C but did not result in TBE incidence growth, and its rate ranges from 0.54 to 1.65. But when AAAT exceeded 1.4°C, there was a dramatic rise in TBE incidence rate up to 16.

**Figure 10.**
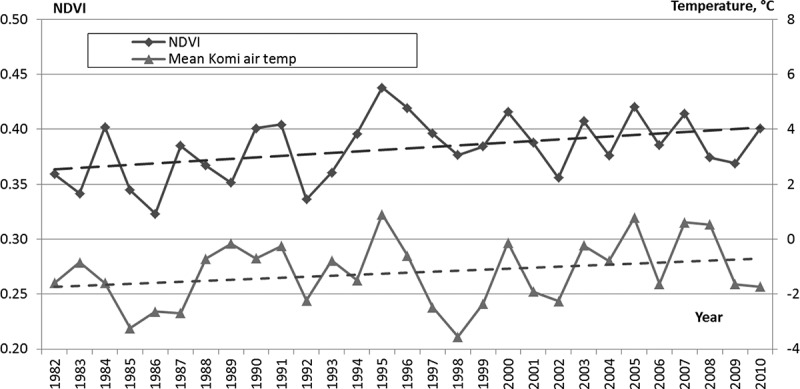
The variations in NDVI and AAAT in RK. Dash lines show linear trends in NDVI and AAAT.

The local NDVI dynamics correspond to AAAT changes and shows a definite increase of vegetation index from 1980s to 2000s (Figure 10). Thus, NDVI in RK increased from 0.36 (in 1980s) to 0.39 (1990s–2000s) when AAAT ran up from −1.57°C to −0.83°C.

## Discussion

In the second half of the twentieth century significant changes in biotic components of the ecosystems occurred in the European North of Russia. There was some northward expansion of forested area, most probably due to climatic changes,[24–26] that caused northward distribution of many species of wild mammals, which are the main tick vectors.

NDVI has been shown to be a good predictor to forecast the abundance of *I*. *ricinus*.[27] In RK NDVI increased with AAAT and confirmed that changes in the local ecosystem improved significantly the life conditions for ixodid ticks over the period under study. Furthermore, in the Arctic region, including the north of Russia, there is an increasing trend in the incidence of some zoonotic diseases not related to ticks.[28,29]

To estimate the changes in tick habitats we analysed TBIR data over 20-year period in different RK districts.

In 1992–1993 it was only in Southern and Central zones (five districts) that people sought medical care after tick bites, however, in 2002–2011 this happened in most RK districts, including those of Northern and Polar zones, even over 65 N°. The same type of health information was given in all zones. Forty years ago the northern boundary of the tick habitat was much closer to the south,[30] but now in RK it has shifted northward by 150–200 km.[31]

The removal of ticks by healthcare providers from residents of Northern and Polar zones who had not travelled out of their district for a at least month, proves that ixodid tick habitat covers now almost the entire territory of RK.

To some extent, the presented data support the assessment of climate-related changes in the range of *I. persulcatus* calculated with the help of simple empirical models linking the possibility of formation a sustainable habitat of ticks to environment temperature and precipitations.[32]

We consider the influence of temperature as a main driving factor of the northward tick expansion in Komi. As the climate of the research area is humid (more precipitation than evaporation) water content does not prevent the spreading of ticks. The distribution of precipitation in Komi is uniform over the year, and there is no dry season.

Tick distribution in new areas, extension of timespan of tick bites, and increase of TBEV prevalence in ticks provide a background for the uptrend in TBE incidence. The northward expansion of tick habitat and TBIR increase are nearly synchronous with the rise in TBE incidence rate and, especially, with that in new territories. This proves that transmission is the main pathway of TBE infection in the RK population. The sharp rise in TBE incidence rate in RK is due both to significant northward shift of the infection and to its considerable increase in the south. Climate change is an important driver of those phenomena. A strong correlation is established between AAAT and TBE incidence rate in Southern, Central and Northern zones and in the whole RK. The correlation degree increases southward, thus indicating that the most pronounced changes both in AAAT and in TBE incidence rate occurred in RK Southern zone synchronously.

The effective temperature governs the duration of the different stages of tick life cycle. The ability of ticks to lay eggs and hatch in one season depends on the sum of effective temperatures.[7,33,34] Complete development of *I. persulcatus* requires a sum of effective temperatures no less than 1,400°C over the period, with a stable average temperature exceeding 10°C.[23] This is only a rough figure only, because with minimal heat provision survival of ticks depends largely on the topography of the land.

In 1970–1997 during the season of tick activity in Southern and Central zones of RK the heat supply only slightly exceeded the critical level (+1.25°C), and did not reach it within Northern zone. In 1998–2011 in Southern, Central, and Northern zones heat supply increased significantly to compare with the earlier period, and in the Northern zone it exceeded the minimal level needed for tick survival. Increased heat supply has improved the life conditions in new territories, resulting in the northward distribution of blood-sucking arthropods.

The northward distribution of ticks due to the increase in air temperature also took place in other north European countries,[35,36] and in the Czech Republic, where TBE-infected ticks were found in the mountains much higher than previously. TBE cases started to occur in those areas.[37–39]

It should be underlined that all above-mentioned studies deal with the spread of TBE by *I. ricinus*. The distinctive feature of our study that it was conducted in the area where *I. persulcatus* dominates. This species is significantly different from *I. ricinus* by its biological characteristics, e.g. due to its much higher cold resistance, so its habitat stretches much to north. Moreover, TBEV prevalence in *I. persulcatus* is usually considerably higher than that in *I*. *ricinus*.[40]

The increasing trend in TBE prevalence in *I. persulcatus*, as revealed in recent years both in questing (flagged) and feeding (on humans and animals) ticks, should be considered as a factor that may have an impact on TBE incidence rate in RK. Moreover, the territory with TBE-infected ticks expanded to the north.

The rise by a few degrees in mean air temperature resulted in a 200–1,000 km northward shift of tick habitat boundaries in Canada.[41]

A sharp rise in TBE incidence caused by the northward distribution of ticks due to climate change had been registered in Arkhangelsk region neighbouring RK,[16] confirming the assumed significant influence of climate change on TBE incidence in the Northern European part of Russia, where there is a boundary of *I. persulcatus* habitat. However, in other Russian regions located further to the south, with permanent abundance of ticks, a rise in AAAT does not trigger TBE incidence growth.[42] This probably means that in localities with temperatures sufficient for ticks some other drivers govern the incidence more.

As it was already mentioned above, climate change in the Northern European part of Russia not only improves the life conditions for *I. persulcatus*, but influences its hosts significantly, providing more favourable conditions for their distribution in new territories. Of course, the higher AAAT may have an impact on the local human population as well, as they spend more time outdoors. This may also increase the probability of exposure to tick bites.[6]

There may be some other factors, apart from climate change, that may have a synergistic effect on the increase in TBE incidence. Evaluation of their roles requires a special investigation.

However, the frequently cited arguments that socio-economic factors,[43–45] increasing awareness of the health authorities [46] or more frequent contacts with ticks due to large-scale agricultural activities [12] contribute to the TBE incidence rise are not particularly applicable in the case of RK.

First of all, the economic trends in RK do not differ from those in other Russian regions. Moreover, in terms of average income per capita, in 2010 RK occupied a rather high place (12th of 80) among the subjects of the Russian Federation, and its ranking was stable for many years.[47]

Secondly, it would be difficult to explain why TBE diagnostics improved recently only in RK, as the all-Russia TBE incidence rate tended to decline. Third, the vast majority of tick bites in RK occurred in leisure time, in parks, gardens or forests, and were not related to production activities. Also, the RK population decreased during the period under study.

The northward distribution of ticks in Northern Europe is a new formidable challenge for the healthcare of northern countries. The severity of the situation is increased by a number of circumstances. Firstly, the majority of *I. persulcatus* ticks in RK are infected with TBEV of Far East genotype, known for its high mortality rate.[48] We cannot exclude the possibility of distribution of this TBE genotype to other countries, e.g. by migrant birds.[49, 50]

Secondly, in the Northern European Russia *I. persulcatus* ticks are infected not only with TBE, but with some other pathogens that cause dangerous diseases.[51–53]

Third, the indigenous population, previously never exposed to tick bites, may be more susceptible to tick-borne infections than the population in RK Southern zone.[15]

These findings show that it is important to improve the prevention of tick-borne infections among local population, especially indigenous Arctic ethnic groups never previously exposed to ticks.

## Conclusions

1. The northward expansion of the *I. persulcatus* range in the Russian Arctic and, particularly, in RK is demonstrated.
2. The reason for this expansion was significant increase both in the average annual air temperature and the sum of effective temperatures that define satisfactory temperature conditions for *I. persulcatus* egg-laying and hatching during one season.
3. Using satellite-provided data it was established that over the past decades in the Russian Arctic, including RK, significant changes in ecosystems took place that improved the life conditions for ixodid ticks.
4. The marked rise in the TBE incidence rate in RK is largely associated with the expansion of the range of *I. persulcatus*. The territory with recorded TBE cases expanded northward significantly. Climate change is an important driver of the increase in TBE incidence.
5. The occurrence of TBE cases in new territories justifies the need to update TBE-preventive measures in current conditions, taking into account the protection of indigenous Arctic ethnic groups.
